# The influence of N-terminal acetylation on micelle-induced conformational changes and aggregation of α-Synuclein

**DOI:** 10.1371/journal.pone.0178576

**Published:** 2017-05-31

**Authors:** David Ruzafa, Yuriko S. Hernandez-Gomez, Giovanni Bisello, Kerensa Broersen, Bertrand Morel, Francisco Conejero-Lara

**Affiliations:** 1 Departamento de Química Física e Instituto de Biotecnología, Facultad de Ciencias, Universidad de Granada, Granada, Spain; 2 Nanobiophysics Group, MIRA Institute for Biomedical Technology and Technical Medicine, Faculty of Science and Technology, Universiteit Twente, Enschede, The Netherlands; Louisiana State University Health Sciences Center, UNITED STATES

## Abstract

The biological function of α-Synuclein has been related to binding to lipids and membranes but these interactions can also mediate α-Synuclein aggregation, which is associated to Parkinson’s disease and other neuropathologies. In brain tissue α-Synuclein is constitutively N-acetylated, a modification that plays an important role in its conformational propensity, lipid and membrane binding, and aggregation propensity. We studied the interactions of the lipid-mimetic SDS with N-acetylated and non-acetylated α-Synuclein, as well as their early-onset Parkinson’s disease variants A30P, E46K and A53T. At low SDS/protein ratios α-Synuclein forms oligomeric complexes with SDS micelles with relatively low α-helical structure. These micellar oligomers can efficiently nucleate aggregation of monomeric α-Synuclein, with successive formation of oligomers, protofibrils, curly fibrils and mature amyloid fibrils. N-acetylation reduces considerably the rate of aggregation of WT α-Synuclein. However, in presence of any of the early-onset Parkinson’s disease mutations the protective effect of N-acetylation against micelle-induced aggregation becomes impaired. At higher SDS/protein ratios, N-acetylation favors another conformational transition, in which a second type of α-helix-rich, non-aggregating oligomers become stabilized. Once again, the Parkinson’s disease mutations disconnect the influence of N-acetylation in promoting this transition. These results suggest a cooperative link between the N-terminus and the region of the mutations that may be important for α-Synuclein function.

## Introduction

Parkinson’s disease (PD) is the most common neuronal motor system disorder affecting more than 1% of the population aged over 65 [1]. PD is characterized by the loss of dopaminergic neurons in the “*substantia nigra*” and the appearance of intraneuronal inclusions known as Lewy bodies, which are composed principally by fibrillar amyloid aggregates of proteins. α-Synuclein (aS) is the major component in these aggregates [2]. A genetic link between PD and aS was also established with the discovery of several missense mutations, corresponding to A30P, E46K and A53T substitutions, in the gene SNCA encoding for aS in kindreds with autosomal-dominantly inherited, early-onset PD [3–5]. Other pathogenic mutations have also been reported more recently [6,7]. A variety of environmental and physiological factors can induce aS self-assembly into oligomers and aggregates, some of which are highly neurotoxic, leading to PD and other neurodegenerative disorders [8,9].

aS is a 140-residue protein widely expressed in brain cells and particularly abundant at the presynaptic terminals of neurons [10]. Its sequence comprises a N-terminal domain, which contains seven imperfect 11-residue lipid-binding repeats, and a C-terminal highly acidic tail. Accumulating evidence relates aS function to a variety of interactions with lipids and membranes. This protein seems to modulate presynaptic vesicle pool size and vesicle recycling and participates in the regulation of synaptic plasticity and dopamine neurotransmission [11]. It also participates in the transport and regulation of polyunsaturated fatty acids (PUFAs) in dopaminergic neurons [12,13], sequestration of arachidonic acid in exocytosis regulation [14], and induction of curvature and remodeling of membranes [15–17].

Although native soluble aS has been considered for many years to be an intrinsically disordered protein [18,19], it acquires α-helical structure upon binding to membranes [20], micelles [21,22] or PUFAs [23,24]. This suggests an important role for this helical structure in aS function. Diverse studies with model micelles and membranes have described that, depending on the surface curvature, aS can adopt different α-helical conformations in its membrane bound state, including an extended helix [20,25], a curved helix [26], or a helix-turn-helix hairpin conformation [21,27]. On the other hand, the influence of lipid interaction on aS aggregation is complex and whether lipids and membranes favor or inhibit formation of toxic oligomers and amyloid fibrils depends on the aS/lipid proportion and the environmental context [28–30].

The anionic surfactant sodium dodecyl sulfate (SDS) has been widely used in biophysical studies to mimic lipid interactions with proteins and to study its effects on protein aggregation [31–33]. Previous studies have shown that SDS can strongly enhance aS fibrillation at relatively low SDS/aS ratios, whereas formation of α-helical structure and interaction with bulk SDS micelles protects from fibrillation [34–36].

The N-terminal region of aS has been described to play a crucial role in the interaction of aS with lipid membranes [37,38]. N-terminal acetylation increases helical propensity of the N-terminal region of aS in its free monomeric state [39]. It also enhances helical structure of aS in the bound state to SDS micelles or negatively charged lipid micelles, as well as affinity for physiological membranes [40,41]. In addition, it has been observed that N-acetylation reduces the aS fibrillation rate by *in vitro* incubation in buffer under shaking conditions [39,42], as well as in the presence of lipid vesicles containing GM1 ganglioside [40]. Despite the evidence that N-acetylated aS (Ac-aS) is universally present *in vivo*, in both the soluble and insoluble fractions of brain tissues of PD patients [43], most *in vitro* biophysical studies of aS aggregation and micelle and membrane interaction have been made with recombinant non-acetylated aS. It is therefore important to increase our understanding of how aS modifications such as N-acetylation and early-onset PD mutations affect the interplay between aS conformational changes, lipid interactions oligomerization and aggregation propensity.

Here we used a variety of biophysical methods to analyze the effects of the interactions with SDS on the structure, oligomerization and amyloid aggregation of aS and Ac-aS. We show that both aS and Ac-aS variants bind and stabilize diverse micellar SDS-aS complexes. In this process, the proteins acquire several interconverting conformational/oligomerization states at different SDS/protein ratios. N-acetylation and early-onset PD mutations alter significantly the equilibrium between these states in different ways, changing the propensity of aS to oligomerize and aggregate. The results suggest the existence of a cooperative link between the N-terminus and the regions of PD mutations than may play an important role for aS function.

## Materials and methods

### Protein production and purification

The different aS variants were produced by overexpression in *E*.*Coli* modified with the plasmid containing the respective sequences. To obtain the respective N-acetylated form, the N-terminal acetyl-transferase B (NatB) plasmid was added to the cells together with 5 μg/mL chloramphenicol to the culture media [44]. The cells were cultured in LB media in the presence of 100 μg/mL of ampicillin at 37°C with shaking until an optical density at 600 nm of 0.6–0.8. Protein expression was induced by adding isopropyl 1-thio-β-D-galactopyranoside to a final concentration of 50 μg/mL (0.2 mM) for 4 h. The cells were harvested by centrifugation at 2200 g for 10 min at 4°C. The pellets were resuspended in buffer (25 mM Tris, 1 mM EDTA, 0.1 mM DTT, pH 7.4, 1 x protease inhibitor mixture (Roche, Penzberg, Germany) and lysed by ultrasonication. The lysate was ultracentrifuged at 105000 g for 45 min at 4°C. The supernatant was filtered (0.45 μm) and applied onto a HiTrap QFF anion-exchange column (GE Healthcare, Chicago, Illinois) previously equilibrated in buffer (50 mM Tris, 0.1 mM DTT, pH 7.4). The protein was eluted using a NaCl gradient of (0–500 mM). After analysis by SDS-PAGE, the pooled protein fractions were precipitated with ammonium sulfate at 47.5% of saturation and the suspension was ultracentrifuged at 105000 g for 30 min at 4°C. The precipitate was resuspended (50 mM Tris, 50 mM NaCl, 0.1 mM DTT, pH 8.3, containing 8M urea) and injected onto a HiLoad 26/60 Superdex 200 column (GE Healthcare, Chicago, Illinois). Subsequently, the protein fractions were pooled and dialyzed extensively against the same buffer in the absence of urea. Then, the protein was concentrated (Amicon ultracentrifugal filter units MWCO 3.000, Millipore, Billerica, Massachusetts) and injected onto a HiLoad 26/60 Superdex 75 column (GE Healthcare, Chicago, Illinois). Pure pooled fractions were dialyzed against 20 mM HEPES, 0.05% NaN_3_, pH 7.2, lyophilized and kept frozen at −20°C. The purified proteins were >95% pure according to SDS-PAGE. The identity of each protein was assessed by electrospray mass spectrometry.

### Circular dichroism

Far-UV CD measurements were performed at 25°C with a Jasco J-715 spectropolarimeter (Tokyo, Japan) equipped with a thermostatic cell holder in a 0.2 mm path length cuvette. The protein concentration was 100 μM and SDS was added from a stock solution of 100 mM to a final concentration ranging between zero and 10 mM. The CD spectra were the average of 5 scans using a bandwidth of 1 nm and a scan speed of 100 nm/min. Baseline spectra were measured with buffer containing each SDS concentration and subtracted from the sample spectra. Data were normalized as mean residue ellipticity and the α-helix content was obtained using its value at 222 nm as described previously [45].

### Dynamic light scattering

DLS measurements were performed at 25°C using a DynaPro MS-X instrument (Wyatt Technology Corporation, Santa Barbara, CA, USA) in a thermostated 30 μL quartz cuvette. The protein solutions and the buffer were filtered through 0.02 μm Anotop 10 filters (Whatman plc, Brentford, Middlesex, UK) before the measurements. Sets of DLS data were acquired every 5 s until at least 20 sets of data were obtained. The measurements were performed in triplicate. Dynamics V6 software (Wyatt Technology Corporation, Santa Barbara, CA, USA) was used in data collection and processing. The experimental autocorrelation curves were analyzed to obtain the particle size distributions using the implemented regularization fit.

### Size-exclusion chromatography

SEC experiments were carried out at room temperature on a Superdex 200 10/300 GL column (GE Healthcare, Chicago, Illinois) at a flow rate of 0.5 mL/min, equilibrated in 20 mM HEPES, 0.05% NaN_3_, pH 7.2, including the appropriate SDS concentration. The column was first calibrated with protein standards of different molecular weight. The aS and Ac-aS samples were freshly prepared in the same buffer, containing the desired SDS concentration at a protein concentration of 100 μM and filtered through 0.02 μm Anotop 10 filters (Whatman plc, Brentford, Middlesex, UK).

### Measurement of aggregation rates by thioflavin T fluorescence

Amyloid aggregation was monitored at 37°C by thioflavin T (ThT) fluorescence using a Varian Cary Eclipse spectrofluorimeter (Agilent Technologies, Santa Clara, CA, USA) equipped with a Peltier-controlled thermostatic cell holder. Protein samples at a final concentration of 100 μM were freshly prepared in 20 mM HEPES, 0.05% NaN_3_, pH 7.2, containing 10 μM ThT and the appropriate SDS concentration. To avoid evaporation during the experiments, the cuvette was sealed using a specific stopper.

### NMR spectroscopy

NMR experiments were performed on a Varian Direct Drive 600 MHz spectrometer (California, USA) at 25°C. For each experiment samples were freshly prepared in deuterium oxide at a protein concentration of 100 μM in 5 mM sodium phosphate buffer, 0.05% sodium azide, pH 7.2, containing the desired SDS concentration. Diffusion-ordered spectroscopy (DOSY) spectra were obtained using a gradient length of 3 ms and a diffusion delay of 250 ms. Gradient strength was previously calibrated using the HDO signal in a doped D_2_O standard. The DOSY data sets were composed by 30 gradient strengths with 128 scans every increment. The DOSY data were processed using MestreNova 10.0 (Mestrelab Research S.L, Spain). Diffusion coefficients were obtained by fitting the intensity decays versus the gradient strength as described elsewhere [46]. The hydrodynamic radii were calculated with the Stokes-Einstein equation, R_h_ = k_B_T/6πηD, where k_B_ is the Boltzmann constant, T is the absolute temperature, D is the diffusion coefficient and η is the viscosity of D_2_O at 298 K.

### Transmission electron microscopy

The morphology of aS aggregates was studied using a Carl Zeiss LEO 906E transmission electron microscope (Zeiss, Oberkochen, Germany). Freshly prepared protein samples were previously incubated at 37°C for different time periods in the presence of several SDS concentrations. 10 μL of the incubated protein solution were left to adsorb on a Formvar 300-mesh copper grid (ANAME, Madrid, Spain) for 1 minute. Then, the sample was negatively stained with 10 μL of 1% (w/v) uranyl acetate solution for 1 minute without previous washing steps. Excess stain was removed with a tissue paper and the grids were air dried before visualization. The samples were observed at a magnification between 60000× and 100000×.

## Results

### Conformational transitions of aS induced by SDS

**Fig 1:**
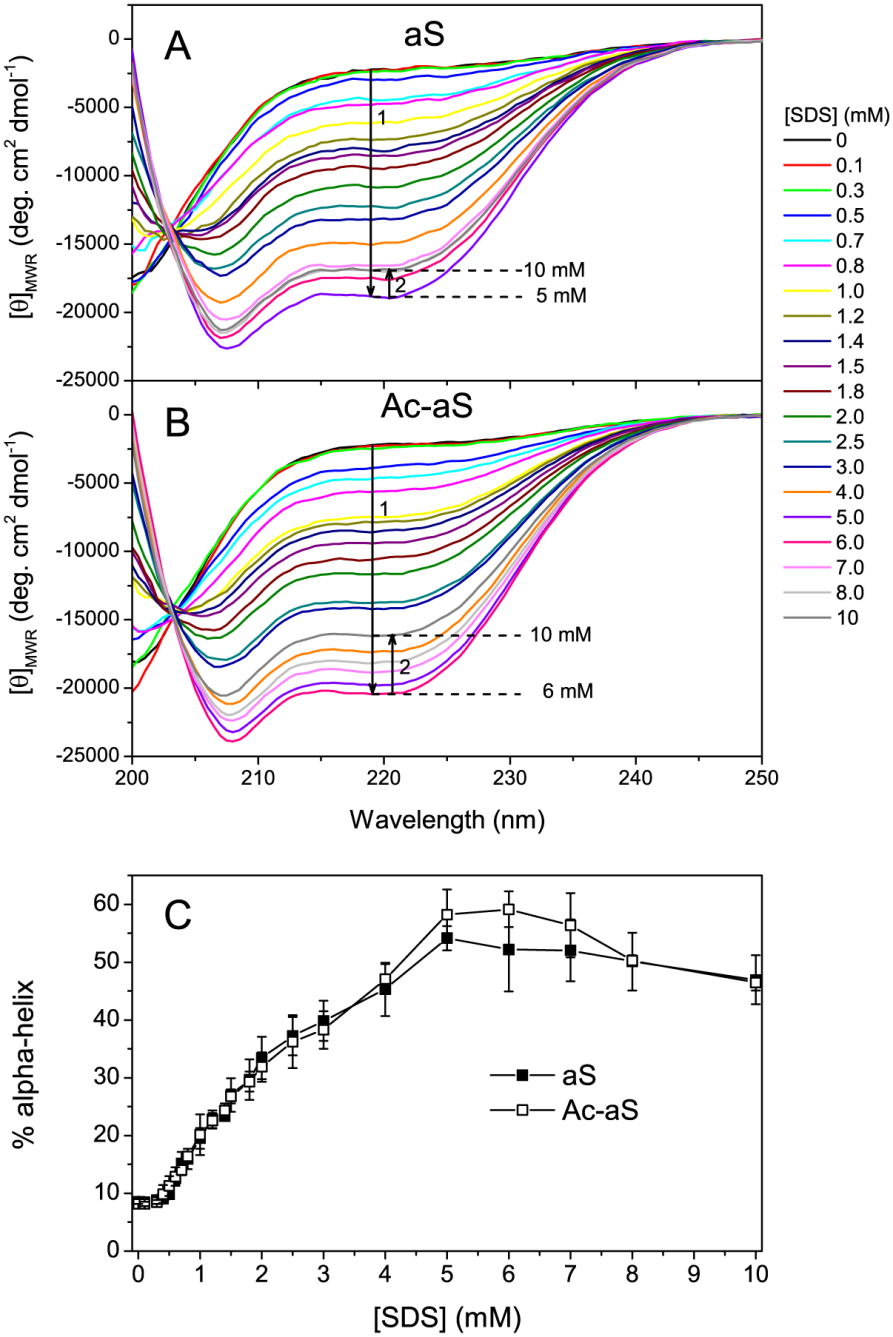
Effect of SDS on the secondary structure of aS and Ac-aS. (A and B) Far-UV CD spectra were recorded at 25°C in 20 mM HEPES buffer pH 7.2, in presence of increasing SDS concentrations for aS (A) and Ac-aS (B). The protein concentration was 100 μM in all measurements. The spectra were normalized to mean residue molar ellipticity. (C) Percentage of α-helix structure of 100 μM aS (filled symbols) and Ac-aS (open symbols) at different SDS concentrations. The values have been calculated from the mean residue ellipticity at 222 nm measured in the far-UV CD spectra [45]. The error bars correspond to at least two independent measurements.

The structural changes of 100 μM wild-type (WT) aS and Ac-aS induced by SDS were observed by CD spectroscopy at 25°C varying the SDS concentration from zero to 10 mM. Under the conditions of this study (20 mM HEPES, pH 7.2, 25°C), the critical micellar concentration (CMC) of SDS was determined by scattering measurements to be 6.4 ± 0.6 mM (S1 Fig), analogous to the value of 7.5 mM reported elsewhere under similar conditions but pH 7.0 [34]. We chose this low ionic strength buffer in order to attain a relatively wide sub-CMC SDS concentration range, since previous studies have shown that binding of helical aS to bulk SDS micelles protects it from aggregation [36]. Below 0.3 mM SDS the two aS variants are mostly unstructured as previously observed, with an estimated 8% α-helix, indicating some α-helical propensity (Fig 1). Above 0.4–0.5 mM SDS there is a gradual formation of α-helical structure produced by SDS. The data suggest three structural transitions. First, between 0.4 mM and about 3 mM SDS the α-helix percent increases gradually to about 40%. In this SDS concentration range the changes are very similar for aS and Ac-aS. Second, between 3 and 5 mM SDS the α-helicity increases slightly more steeply and reaches a maximum. This maximum appears slightly more pronounced for N-acetylated aS although the difference is not statistically significant. Third, between 7 mM and 10 mM SDS the α-helical structure decreases again to about 45%. Similar conformational transitions were observed in a previous study for aS [36].

### Formation of SDS-aS oligomeric complexes

**Fig 2:**
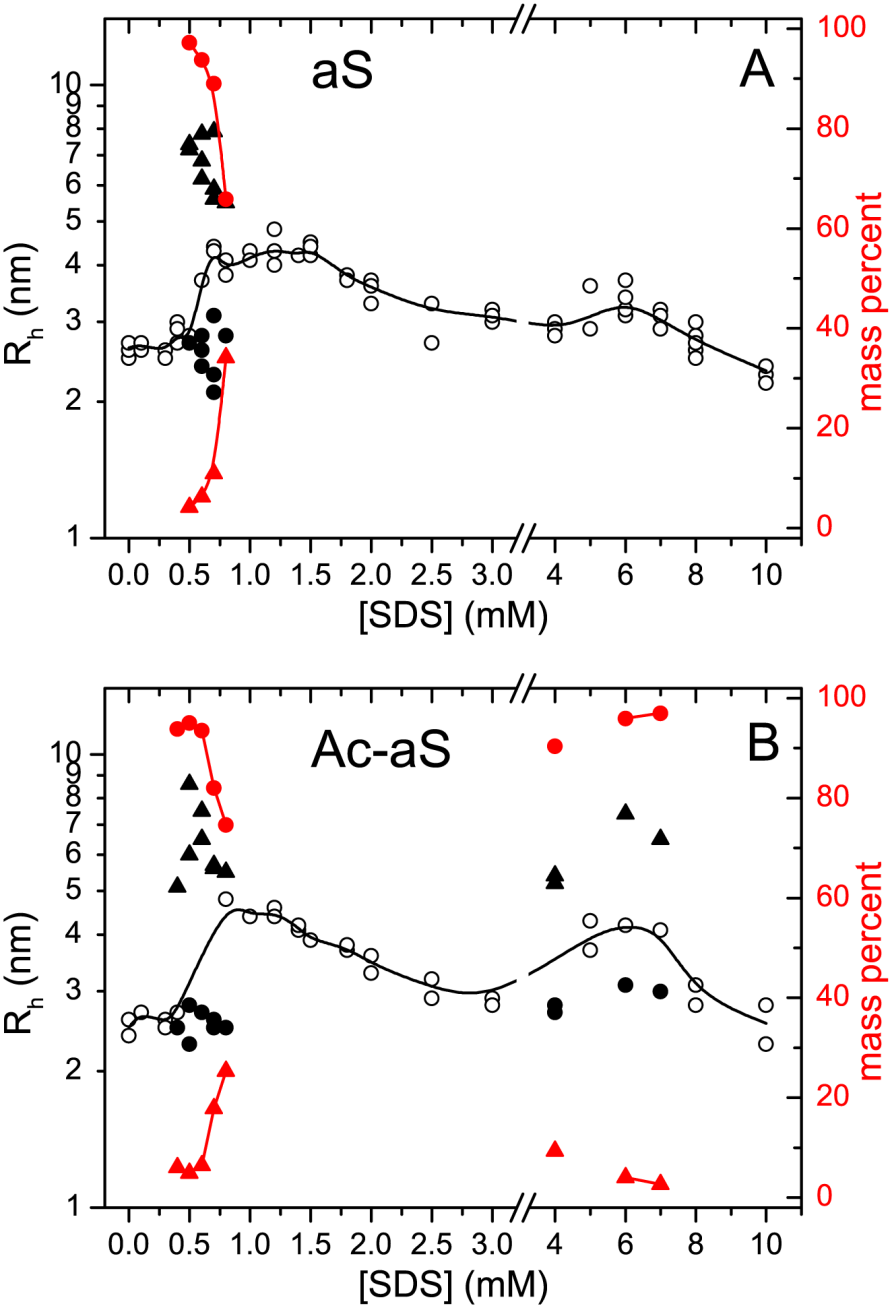
Changes in molecular size of aS (A) and Ac-aS (B) as a result of interactions with SDS. The apparent hydrodynamic radius (R_h_) of 100 μM protein in presence of different SDS concentrations was measured by DLS at 25°C. The black symbols represent the R_h_ of particles (left scale) corresponding to more than 2% of the protein mass and more than 10% of the scattered intensity. Measurements reporting two different particle sizes (circles and triangles) are represented with filled symbols. The black solid line corresponds to the intensity-averaged R_h_ at each SDS concentration. The red symbols represent the mass percent of each type of particle (right scale).

To investigate the changes in molecular size of aS and Ac-aS as a result of their interaction with SDS, we analyzed the mixtures by dynamic light scattering (DLS) (Fig 2). In the absence of SDS, both aS and Ac-aS show identical hydrodynamic radius (R_h_) of 2.6 ± 0.1 nm, in agreement with compact disordered aS [47]. Between zero and 0.3 mM SDS the apparent R_h_ does not change significantly for most of the protein mass and only trace amounts of oligomers and large aggregates could be detected. Between 0.4 mM and 0.8 mM SDS, a significant and increasing fraction of the proteins form particles with apparent R_h_ ranging between 5 and 9 nm, which may be attributed to SDS-associated aS oligomers. These oligomers could be discerned from the aS monomers only in some of the DLS measurements, whereas in others the two different sizes could not be resolved, resulting in an averaged R_h_. This may be due to a limited resolving power of DLS for these size differences but is also possible that the time scale of DLS (μs to ms) may be close to that of this oligomerization equilibrium. The fraction of protein mass that forms oligomers increases rapidly for both variants with a rise in SDS concentration, with concomitant reduction in the monomer fraction. From 1 mM to 3 mM SDS, a single average R_h_ value could only be measured for both proteins, reaching about 4.5 nm between 0.8 mM and 1.5 mM SDS and then decreasing gradually to about 3 nm at ∼3 mM SDS for Ac-aS and at ∼4 mM for aS. This suggests a reduction in the number of aS molecules in each SDS-aS complex but it could also indicate a more compact conformation of aS in the bound state, as a result of a higher α-helical content, as observed by CD. In fact, the average α-helix content of both Ac-aS and aS increases up to about 40% at these SDS concentrations.

More prominent differences between the two protein variants were found in the 4–10 mM SDS concentration interval. A second increase in the apparent R_h_ takes place between 3 and 6 mM SDS for Ac-aS. This is again a result of exchange between two types of particles of different sizes that could be resolved in some DLS measurements. A maximum average R_h_ of 4 nm is observed for Ac-aS between 5 and 6 mM SDS, coincidently with the maximum α-helical percentage, and is followed by a further R_h_ decrease to about 2.5 nm at the highest SDS concentration investigated (10 mM). These results indicate that self-association of protein-SDS complexes to form larger particles involves an additional α-helical conformational change in Ac-aS. This second oligomerization process is considerably less pronounced for non-acetylated aS.

### Free SDS and aS are in rapid exchange with SDS-aS oligomeric complexes

**Fig 3:**
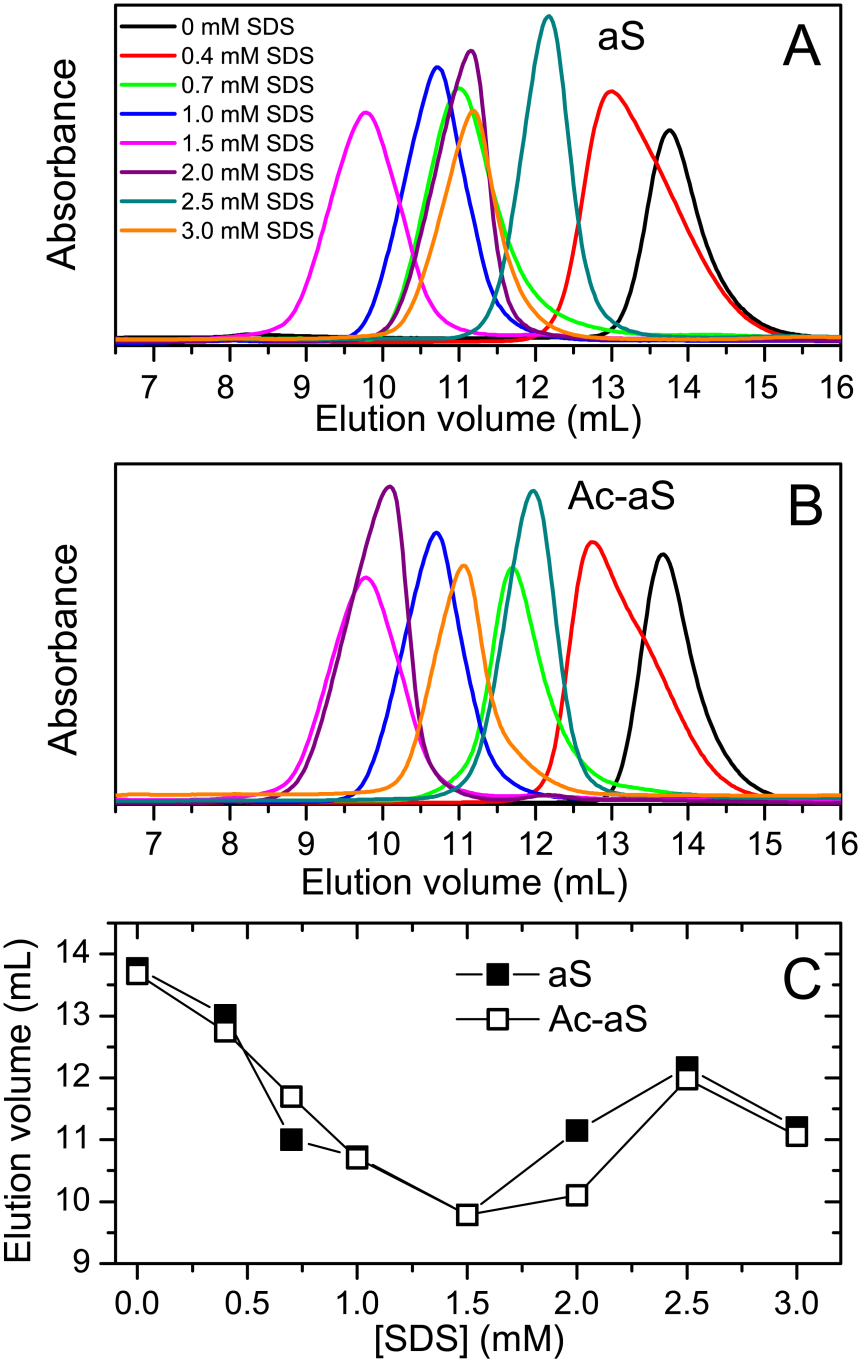
Hydrodynamic properties of aS and Ac-aS. Size-exclusion chromatography analysis aS (A) and Ac-aS (B) in presence of different SDS concentrations, as indicated in different colors. (C) Elution volumes of each peak maximum at each SDS concentration.

The protein-SDS mixtures were also analyzed by size-exclusion chromatography (SEC) at different SDS concentrations (Fig 3). Unfortunately, the SDS concentration could be increased only up to 3 mM because SDS interacted with the column matrix at higher concentrations. In the absence of SDS, both monomeric aS and Ac-aS elute at volumes corresponding to a 36 kDa globular protein, in good agreement with the apparent hydrodynamic radii measured by DLS corresponding to a compact disordered protein. At 0.4 mM SDS a considerable peak broadening could be observed, suggesting exchange between the free monomer and SDS-bound protein oligomers. At higher SDS concentrations the proteins eluted at lower elution volumes and always as single peaks indicating fast exchange between aS monomers and aS-SDS oligomers. When aS was preincubated with SDS and then analyzed in pure buffer the protein eluted as monomer (not shown), which confirms that the SDS-protein interaction is rapidly reversible. No evidence of large aggregates eluting in the exclusion volume was observed under these conditions. The elution volumes changed in parallel for both proteins with the SDS concentration. The lowest elution volume was observed at 1.5 mM SDS corresponding to that of a globular protein of about 220 kDa.

**Fig 4:**
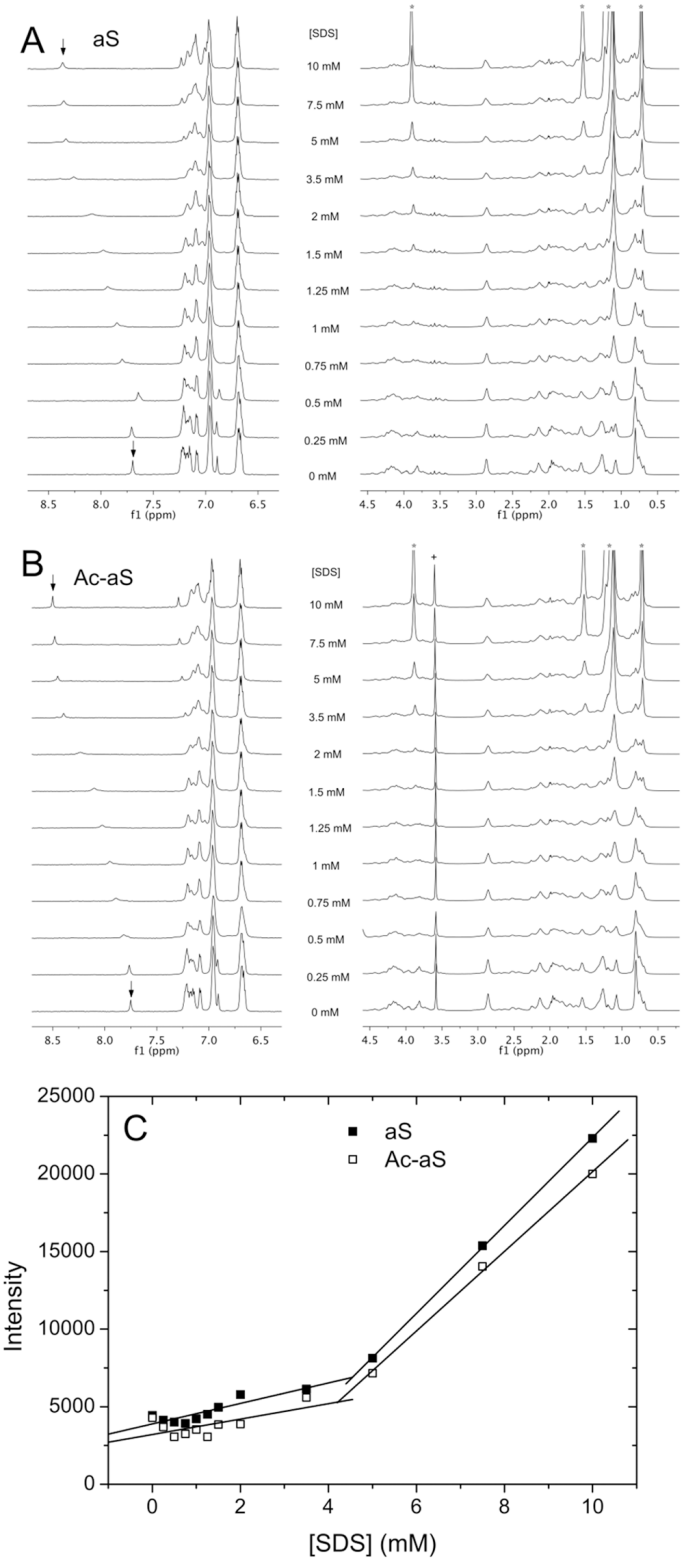
Effect of SDS on the NMR spectra of aS and Ac-aS. Aromatic and aliphatic regions of the 1D ^1^H NMR spectra of aS (A) and Ac-aS (B) in 100% D_2_O, 5 mM sodium phosphate buffer pH 7.2, in the presence of different SDS concentrations. Spectra were acquired at 25°C at 100 μM protein concentration. The intensities of each spectral region have been adapted for proper visualization. The H_ε1_ resonance of the His50 is identified with arrows at 0 and 10 mM SDS. The signal marked with a cross corresponds to an impurity. The positions of the free SDS resonances have been marked with asterisks. (C) Signal intensity at the position of the resonance of α-methylene protons of free SDS at different SDS concentrations in presence of 100 μM aS and Ac-aS. The intensity values correspond to the integral of the spectra between 3.80 and 3.95 ppm.

1D ^1^H-NMR spectra of the two aS variants were recorded at 25°C at different SDS concentrations. To avoid interference from the intense resonances from the HEPES buffer, we used 5 mM sodium phosphate buffer pH 7.2 in 100% D_2_O for these experiments. The ionic strength of this buffer (about 0.014 M) is similar to that of the HEPES buffer used in the rest of the experiments. Many protein resonances become broadened at SDS concentrations between 0.25 mM and 3.5 mM and sharpened again above 5 mM SDS (Fig 4). Particularly evident is the behavior of the well-resolved H_ε1_ resonance of the His50 side chain, which is considerably broadened and gradually downfield shifted between 7.7 and 8.5 ppm, as a result of the interactions with SDS. This is consistent with a moderately rapid exchange in the NMR time scale between the free protein and SDS-aS oligomeric complexes. The conformational change sensed by His50 is complete above 5 mM SDS. Another interesting observation is that below 2 mM there are no visible sharp resonances attributable to free monomeric SDS, as a result of strong line broadening. Above 3.5 mM the free SDS resonances start to emerge in the spectra, although still broadened, and their intensities increase linearly with the total SDS concentration. Signal integration around the position of the free SDS signals shows two separate trends with a sharp break around 4.5 mM (Fig 4C). These results indicate a maximum binding stoichiometry of about 40–50 SDS molecules per aS molecule, similar to that reported elsewhere [36]. At higher concentrations, SDS is in rapid exchange between SDS monomers, free SDS micelles and SDS-aS micellar complexes.

**Fig 5:**
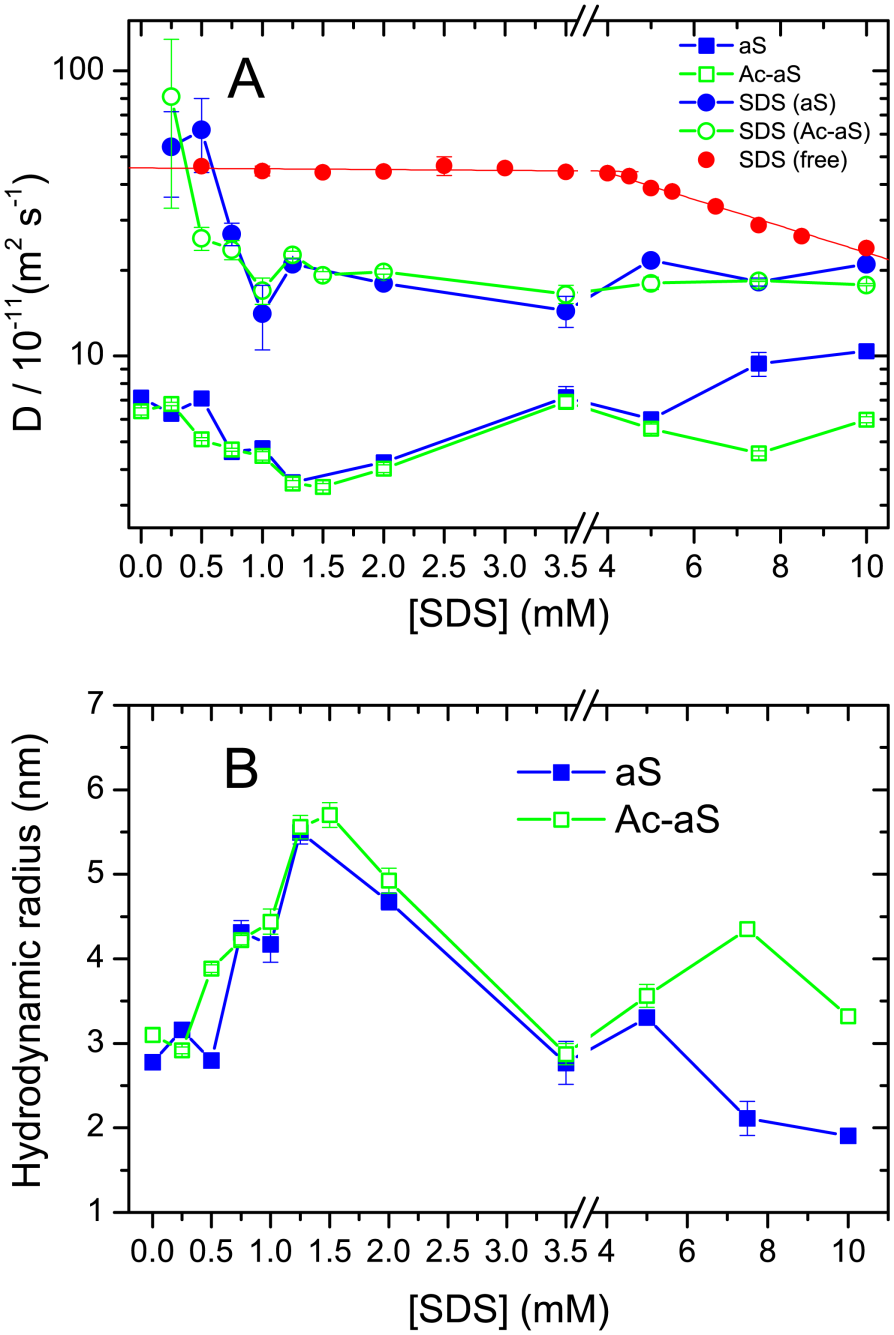
Hydrodynamic properties of SDS, aS and Ac-aS measured by DOSY NMR. (A) Diffusion coefficients of SDS, aS and Ac-aS in free SDS solutions and protein-SDS mixtures at different SDS concentrations. DOSY experiments were carried out at 25°C in 100% D_2_O, 5 mM sodium phosphate buffer pH 7.2. Protein concentration in the mixtures was 100 μM. Errors correspond to 95% confidence intervals of the fittings of the DOSY intensity decays. The CMC of SDS (3.8 mM) was estimated from the intercept of the two observed linear tendencies. (B) Apparent hydrodynamic radius of aS and Ac-aS in presence of different SDS concentrations calculated from the diffusion coefficients shown in (A).

Diffusion-ordered spectroscopy (DOSY) ^1^H-NMR measurements were carried out for each aS variant at 100 μM protein concentration in the presence of SDS from zero to 10 mM. The CMC of free SDS under these conditions was determined as 3.7 ± 0.3 mM by scattering measurements (S1 Fig) and 3.8 mM from DOSY measurements (Fig 5A). The diffusion coefficient of monomeric SDS is (4.6 ± 0.2) × 10^−10^ m^2^ s^−1^, in good consistency with values ranging from 4.7 to 4.9 × 10^−10^ m^2^ s^−1^, as published elsewhere [48]. Above the CMC, monomeric SDS is in rapid exchange with micelles and its diffusion coefficient decreased progressively with an increase in concentration.

Translational diffusion coefficients were measured for aS and Ac-aS by fitting the decay of the intensity of the protein resonances with the gradient strength, using the integrals of spectral regions devoid of the influence of SDS signals, *i*.*e*., the region between 6.5 and 7.5 ppm, corresponding to the aromatic protons, and the region between 1.7 and 3.2, in the aliphatic region. The diffusion coefficients of both proteins are represented in Fig 5A. The apparent hydrodynamic radii derived from these data are plotted in Fig 5B. In the absence of SDS the apparent R_h_ of the two proteins is consistent with their monomeric state. Above 0.5 mM SDS the R_h_ of the two proteins increases to about 5.5 nm at 1.25–1.5 mM SDS and then decreases to about 3 nm at 3.5 mM SDS, consistently with the observations by DLS. However, in contrast to DLS, monomeric and oligomeric particles could not be resolved in the DOSY experiments. The size increase starts at slightly lower SDS concentration for Ac-aS than for aS. At higher SDS concentrations there is another size increase for Ac-aS that is much less pronounced for aS, as it was observed by DLS. This confirms that Ac-aS shows a higher propensity than aS to form oligomeric complexes with SDS in the high SDS concentration range.

To estimate the diffusion coefficient of SDS in the mixtures we used the decays of the integrals of two spectral regions around the SDS signals, *i*.*e*., the region between 4.5 and 3.2 ppm and the region between 1.6 and 0.4 ppm. Since these regions include both SDS and protein resonances, the signal decays were fitted using a double exponential function, fixing the diffusion coefficient of the protein to the values previously obtained from the spectral regions devoid of SDS resonances (S2 Fig). The diffusion coefficient of SDS (Fig 5A) decreases sharply between zero and 1 mM SDS and then fluctuates around 1.5–2.0×10^−10^ m^2^ s^−1^ for the rest of the SDS concentration range. These diffusion coefficients tend to converge with those measured for free SDS above its CMC. These data suggest that above 1 mM a major fraction of SDS becomes incorporated in aS-SDS micellar complexes.

### Rapid formation of amyloid oligomers and fibrils induced by SDS

**Fig 6:**
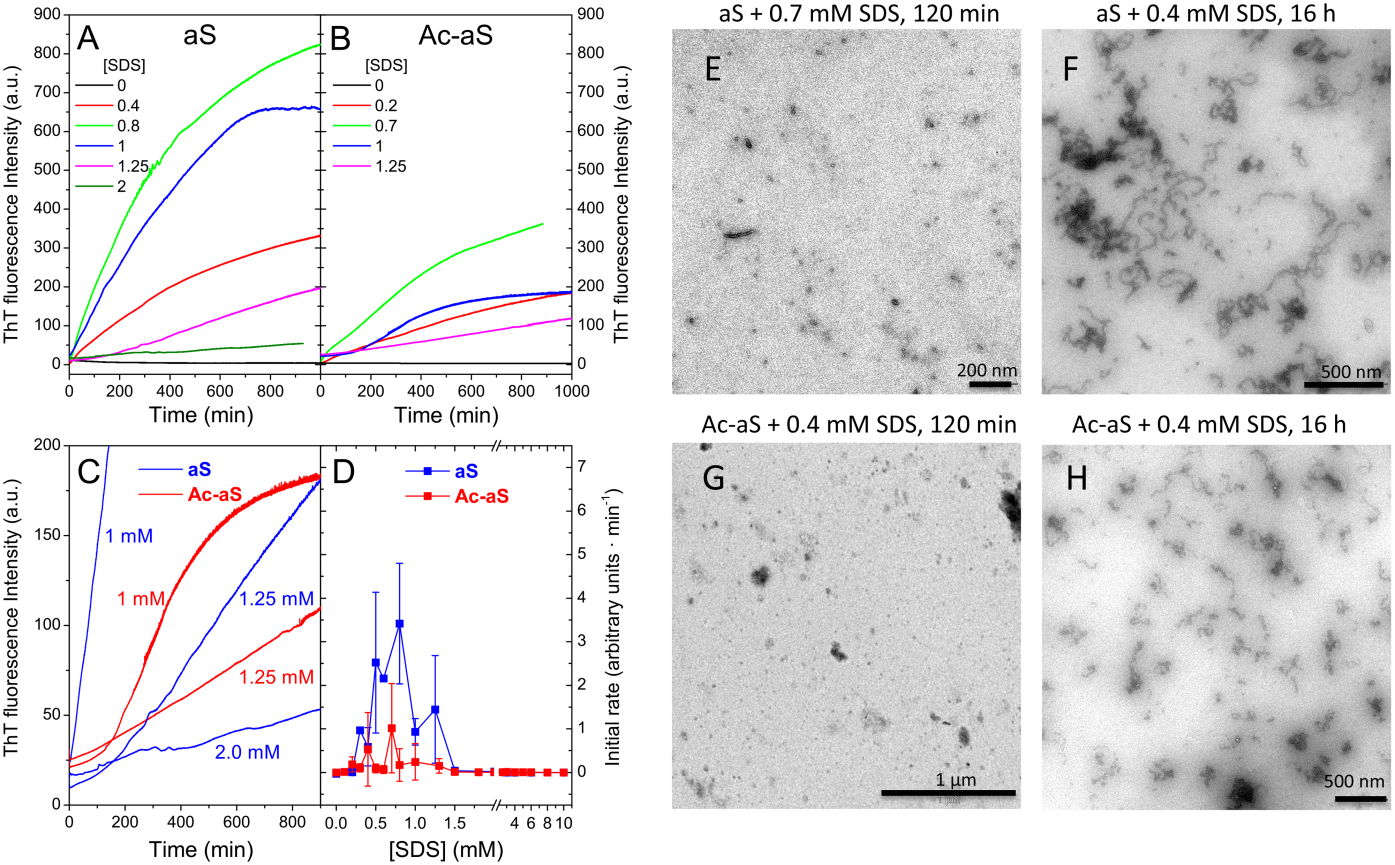
Amyloid aggregation induced by SDS. (A and B) Representative aggregation kinetics of aS (left panel) and Ac-aS (right panel) at 37°C followed by ThT fluorescence in presence of different SDS concentrations. (C) Scale expansion of some of the kinetics showing incipient lag phases. Numbers alongside each trace indicate the SDS concentration for aS (blue) and Ac-aS (red). (D) Initial aggregation rates at 37°C of WT aS and Ac-aS in presence of different SDS concentrations. The rates have been determined from the initial slopes of the ThT fluorescence kinetics. Error bars correspond to standard deviations from several independent measurements. (E-H) Transmission electron microscopy images of SDS-induced aggregates of aS (E and F) and Ac-aS (G and H). Samples at 100 μM concentration were incubated at 37°C in presence of SDS during the times lengths and at the SDS concentrations indicated on top of each image.

To investigate how the N-terminal acetylation influences the aggregation of aS induced by SDS, we monitored the aggregation of both acetylated and non-acetylated aS variants in the presence of different SDS concentrations by thioflavin T (ThT) fluorescence. Aggregation kinetics were measured at 37°C in 20 mM HEPES buffer pH 7.2 at a protein concentration of 0.1 mM under quiescent conditions (Fig 6). In the absence of SDS, neither of aS nor Ac-aS aggregated significantly for several weeks under our experimental conditions. However, low SDS concentrations ranging between 0.2 mM and 1.5 mM induced rapid aS aggregation (Fig 6A–6B). The initial slopes in the kinetics are representative of a rapid nucleation of amyloid structure [49]. The measured initial rates (Fig 6D) show however considerable variability in different sample preparations with identical SDS concentrations, resulting in large uncertainties. Nevertheless, aggregation of non-acetylated aS is significant above 0.3 mM SDS. Maximum aggregation rates occur between 0.5–0.8 mM SDS and then decrease. At 1.25 mM SDS, an incipient lag phase becomes noticeable in the kinetics (Fig 6C). Above 2.5 mM SDS the aggregation rate is negligible. A similar tendency was observed for Ac-aS but the aggregation rates are considerably reduced compared to aS and some lag is perceptible at lower SDS concentrations (Fig 6C). For both protein variants, the maximum aggregation rates occur at SDS concentrations where monomeric aS coexists with large SDS-aS oligomeric complexes, suggesting that both states may contribute to productive formation of amyloid nuclei.

The morphology of the aS aggregates induced by SDS was analyzed by transmission electron microscopy (TEM) (Fig 6E–6H and S3–S5 Figs). At early time points during the aggregation kinetics, only small globular aggregates or clusters of them and few small protofibrils were observed for both proteins. Formation of these aggregates produced a rapid growth of ThT fluorescence, which indicates that they have amyloid structure. After 16 hours of incubation, more protofibrils and curly fibrils appear to develop by association of the globular aggregates. Longer and straighter fibrils grow after several weeks of incubation for both protein variants (S5 Fig). No clear differences in the morphology of the aggregates could be observed between aS and Ac-aS, although the progress of the fibril assembly under each condition seemed to be slower for the acetylated protein, in agreement with the ThT fluorescence experiments.

### Effects of N-acetylation in early-onset PD variants

**Fig 7:**
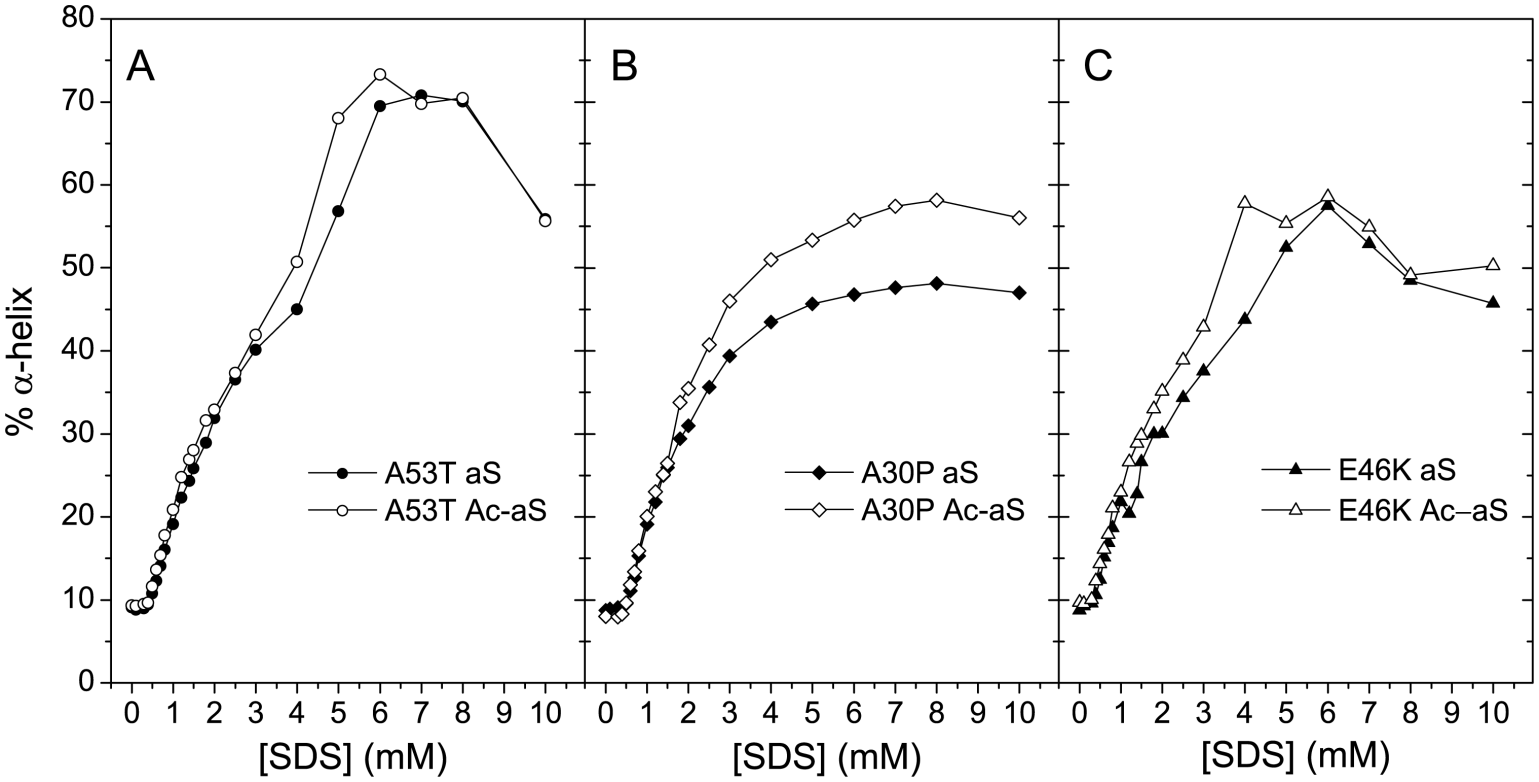
Changes in secondary structure in early-onset PD variants. Percentage of α-helix structure of early-onset PD variants of aS (filled symbols) and Ac-aS (open symbols) at different SDS concentrations. The values have been calculated from the mean residue ellipticity at 222 nm measured in the far-UV CD spectra [45].

We also explored the influence of the early-onset PD mutations A30P, E46K and A53T, on the conformational changes and interactions of aS and Ac-aS induced by SDS by CD and DLS measurements. In the absence of SDS, all variants have a similar disordered structure and practically identical hydrodynamic radii. None of the mutations affects noticeably the growth of α-helical structure in the low SDS concentration range. The structural differences in the high SDS concentration range are however more remarkable. Both the acetylated and non-acetylated A53T variants acquire higher α-helix content between 4 mM and 10 mM SDS than the two WT variants (Fig 7). In contrast, the A30P variants show a more steady growth in α-helix structure than the WT forms and do not display a clear maximum in α-helix between 4 and 10 mM SDS. The E46K variants show a conformational behavior more analogous to the WT proteins.

**Fig 8:**
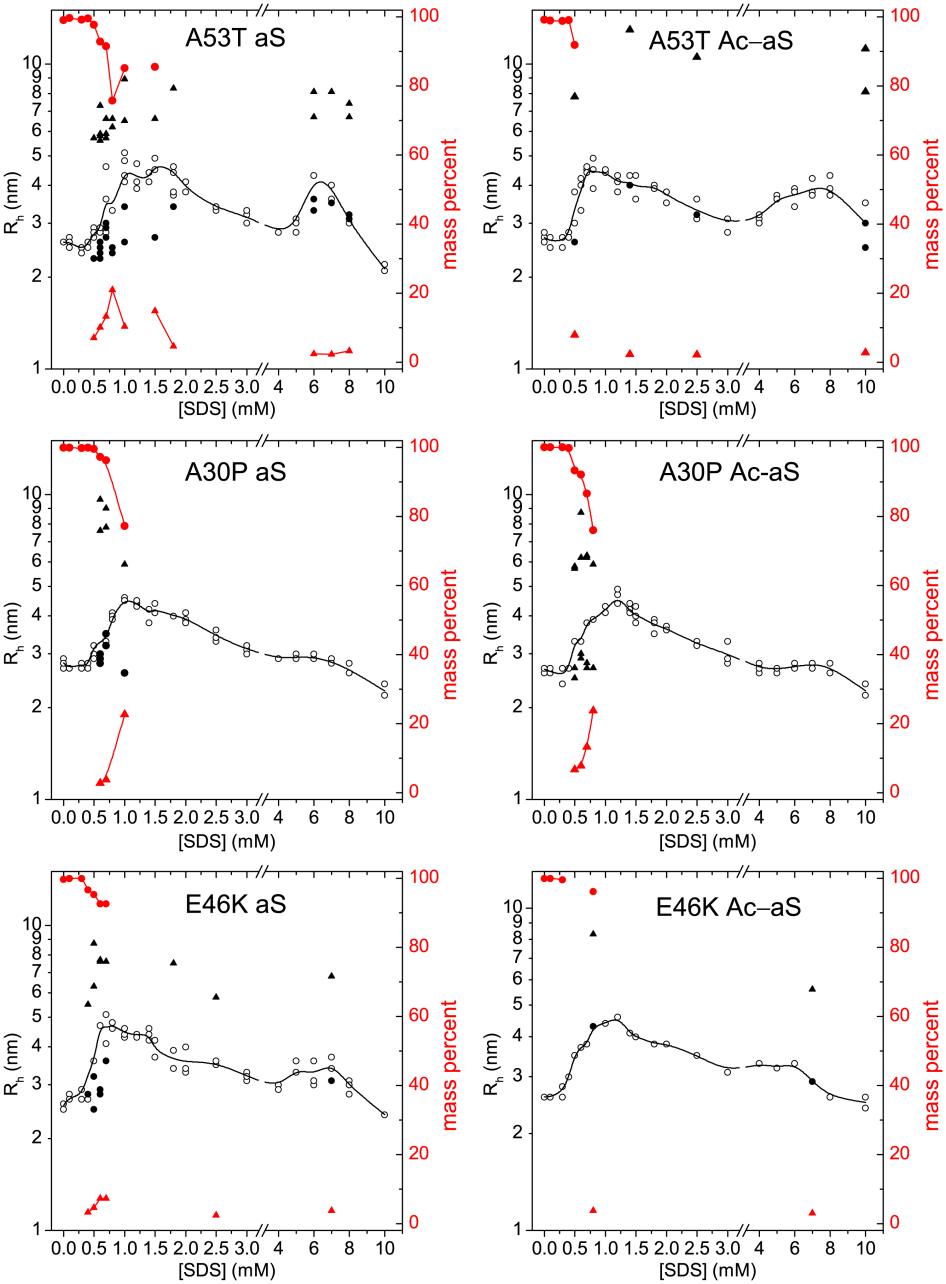
Changes in molecular size of early-onset PD variants. The apparent hydrodynamic radius (R_h_) of 100 μM aS variants (left panels) or Ac-aS variants (right panels) in presence of different SDS concentrations was measured by DLS at 25°C. The open symbols represent the R_h_ of particles (left scale) corresponding to more than 2% of the protein mass and more than 10% the scattered intensity. Measurements reporting two different particle sizes (circles and triangles) are represented in red. The black solid line corresponds to the intensity-averaged R_h_ at each SDS concentration. Closed symbols represent the mass percent of each type of particle (right scale).

According to our DLS analysis, all the PD variants form SDS-aS oligomers similar to the WT proteins between 0.4 mM and 4 mM SDS (Fig 8). The overall evolution of the averaged R_h_ with the SDS concentration is roughly similar for all the aS variants in this SDS concentration range, with only subtle differences. Similarly to the WT variants, at low SDS/aS concentration ratios two different particle sizes could be resolved in some DLS measurements, indicating coexistence of monomeric protein with large oligomeric SDS-protein complexes. This is particularly evident for the non-acetylated A53T aS variant. In contrast, the formation of α-helix-rich oligomers at high SDS concentrations is affected considerably by the mutations. The A53T mutation clearly promotes the formation of this type of oligomeric complexes to a similar extent as the WT Ac-aS, as evidenced by the transient increase in R_h_, coincidently with the high content in α-helix observed for this variant in this concentration range. In contrast, none of the A30P variants showed a size increase in the 4–10 mM SDS concentration range, in agreement with the absence of transient α-helical increase. This indicates that the A30P mutation inhibits this transition irrespective of the presence or absence of N-acetylation. The E46K aS variant shows an intermediate behavior similar to the WT non-acetylated aS but in contrast to the WT protein the N-acetylation of this variant appears to reduce the oligomerization process. This suggests an antagonistic effect between the E46K mutation and the N-acetylation with regards to this oligomerization transition.

**Fig 9:**
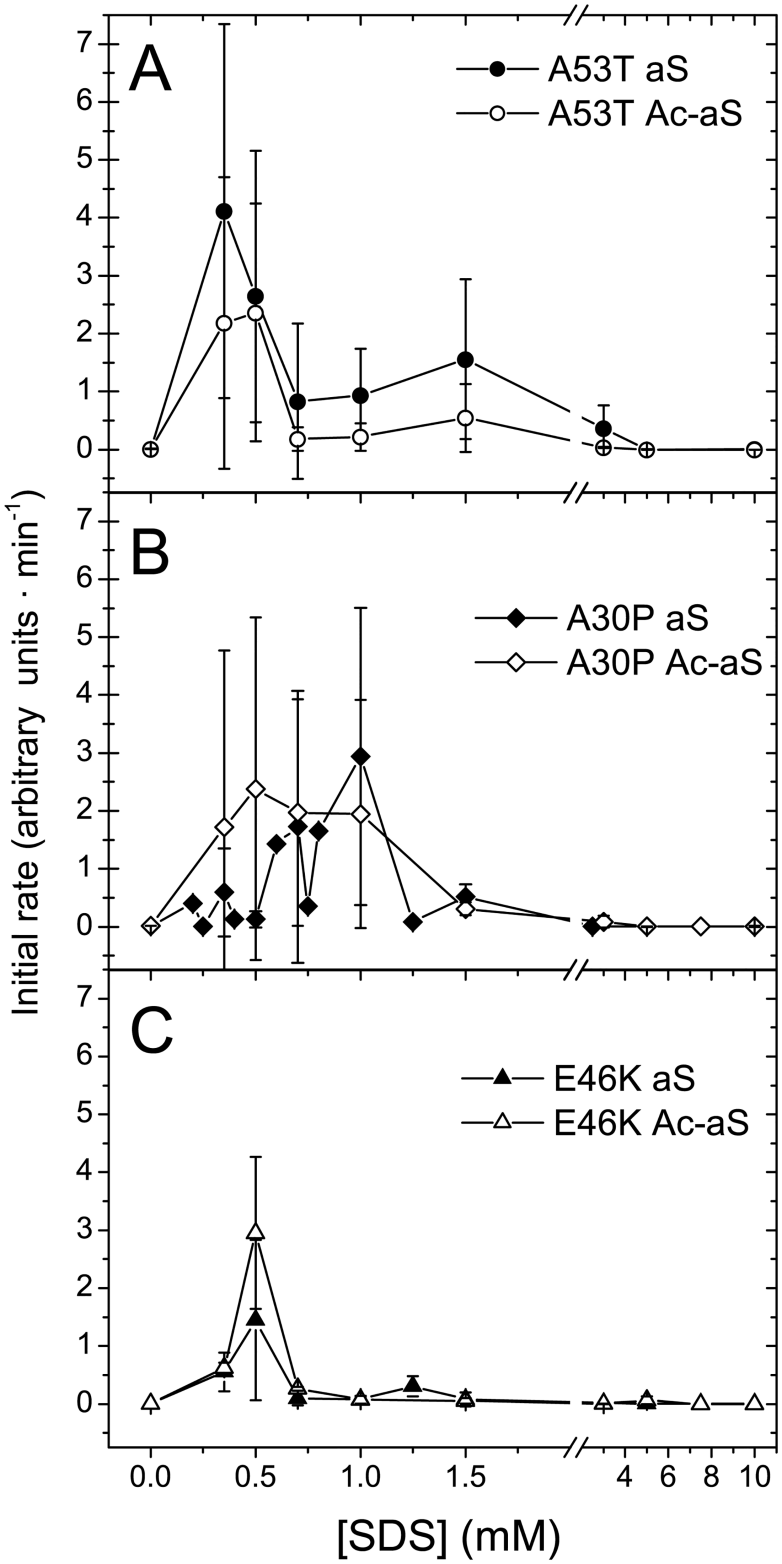
Amyloid aggregation rates of early-onset PD variants. Initial aggregation rates at 37°C of aS and Ac-aS PD variants in presence of different SDS concentrations were measured by ThT fluorescence. The rates were determined from the initial slopes of the ThT fluorescence kinetics. Error bars correspond to standard deviations from several independent measurements.

The aggregation rates of the early-onset PD variants in the presence of SDS were also measured by ThT fluorescence (Fig 9 and S6 Fig). Similar to the WT variants, most the aggregation kinetics show no lag phases but the initial aggregation rates varied considerably in measurements with carefully prepared identical experiments. Nevertheless, overall effects of the mutations could be observed. The maximum aggregation rates measured did not change dramatically between the different variants but the SDS concentration range where significant aggregation occurs was markedly different. The fastest aggregation of the A53T aS variant occurred around 0.3–0.5 mM SDS, a concentration where a majority of the protein is monomeric. However, this variant aggregates over a broad range of SDS concentrations and significant aggregation remains even up to 3 mM SDS. This may be related to the coexistence of large oligomers and aS monomers over a relatively broad SDS concentration range as detected by DLS for the non-acetylated A53T variant (Fig 8). N-acetylation of the A53T variant appears to reduce only moderately the aggregation rates in the high SDS concentration range. The A30P variants aggregate in an intermediate range of SDS concentrations, whereas the two E46K variants only aggregate around 0.5 mM in a much narrower SDS concentration range than the other variants. No significant changes in aggregation propensity due to N-acetylation could be measured for these two variants. These results suggest that the protective effect of N-acetylation against micelle-induced aggregation observed for the WT proteins is considerably impaired for the PD variants.

## Discussion

**Fig 10:**
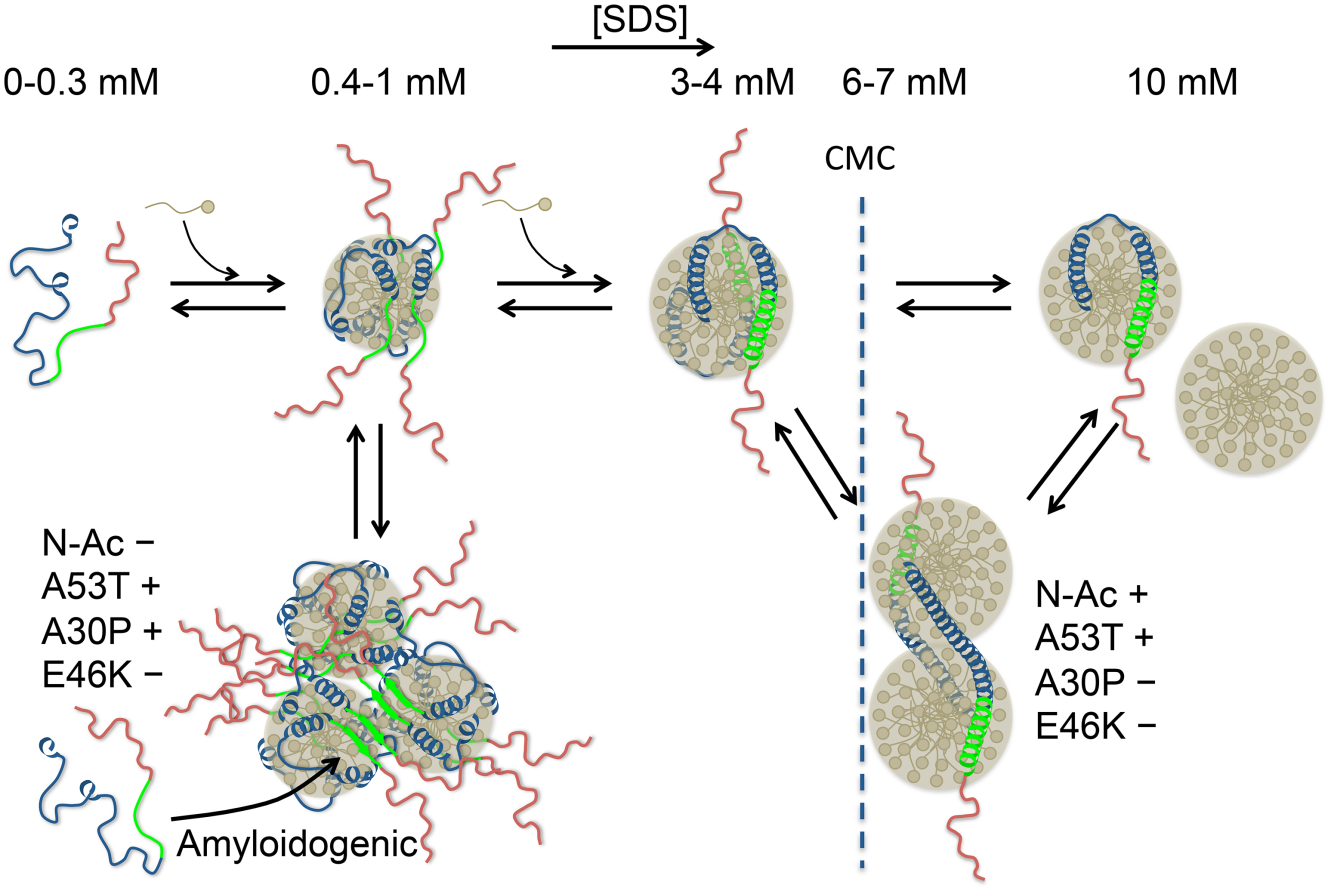
Schematic illustration of the proposed conformational-oligomerization equilibria of aS in the presence of different concentrations of SDS. The N-terminal lipid-binding region of aS is colored in blue, the NAC region in green and the acidic C-terminal tail in red. Spherical SDS micelles have been depicted for simplicity. The effect of N-acetylation and PD mutations on the amyloid aggregation rate and on the stability of α-helix-rich oligomers is indicated with + or − symbols.

In this study we examined in detail the interactions between SDS and aS, both in its N-acetylated and non-acetylated forms. Many observations made in previous studies for WT non-acetylated aS [34,36,50,51] were confirmed here but we provided new evidence highlighting an essential role of oligomerization in a variety of interactions between aS and SDS, as well as in the SDS-mediated aS aggregation. Fig 10 shows a schematic cartoon illustrating the proposed conformational-oligomerization equilibria of aS and SDS at different SDS concentrations and explaining how the interactions with SDS micelles may induce or inhibit aS aggregation depending on the SDS/aS ratio.

### Under an SDS concentration threshold α-Synuclein remains as a compact disordered monomer irrespective of N-acetylation

In a low SDS concentration range (0–0.3 mM) all the aS variants studied are mainly unstructured and monomeric. Our DLS and DOSY data did not detect any size change in either aS or Ac-aS in this SDS concentration range. Moreover, the apparent diffusion coefficient of SDS was not significantly reduced in presence of the proteins at 0.25 mM SDS. Therefore, although monomeric aS may interact with SDS in this concentration range, this interaction is likely weak and unspecific and does not induce a detectable conformational change. In the absence of SDS, N-acetylation has been shown previously to increase the α-helical propensity at the first nine residues in WT aS and produce long-range perturbations at regions 28–31, 43–46 and 50–66 of the sequence [39]. These effects did not produce however any detectable changes in the size or overall conformation when comparing N-acetylated and non-acetylated aS variants. Nevertheless, these long-range links between conformational propensities of the N-terminus and the regions of the early-onset PD mutations have been proposed to be important in the fibrillation propensity of aS in the absence of SDS [52]. This is of interest because we observed high SDS-induced aggregation rates under conditions where a large fraction of aS is still monomeric, suggesting a key role of the monomer as substrate for aggregate growth. The importance of the ratio of monomeric to aggregated forms for productive amyloid fibril growth and neuronal toxicity has also been highlighted for β-amyloid peptide [53].

### Mixed SDS-aS oligomeric amyloidogenic complexes form at low SDS/aS ratios

Above 0.4–0.5 mM and up to 0.8–1 mM SDS, depending on the aS variant, there was a rapid increase in the fraction of aS that becomes associated to SDS. This process resulted in an increase in the average molecular size and in the overall α-helix structure. Our results show that the maximum rate of formation of amyloid aggregates occurs when monomeric disordered aS is in exchange with a significant population of large oligomeric SDS-aS complexes where aS acquires partial α-helical structure. This supports the notion that aS clustering within these complexes may favor the conformational conversion necessary to nucleate the amyloid structure, which further grows by monomer incorporation. Supporting this view there is a recent study showing that rapid aS fibrillation is nucleated on the surface of negatively charged lipid vesicles [54]. This nucleation mechanism becomes enhanced by several orders of magnitude relative to other nucleation mechanisms likely due to a local concentration effect. However, productive aggregation appears to depend also on the availability of aS monomers, as inferred by the decrease in the rates of aggregation at SDS concentrations where monomeric aS is depleted favoring the SDS-aS complexes.

Given the key role of the SDS-aS oligomeric complexes in aggregation it is important to discuss about their nature. Even assuming that a large fraction of SDS is bound to aS under these conditions, a relatively low SDS/aS stoichiometry is conceivable in the oligomers formed between 0.5 and 1 mM SDS. Also, aS still has a low level of α-helix structure (<20%) in this SDS concentration range, which implies a relatively disordered protein in these micellar complexes. Ahmad et al. [34] also reported the formation of fibrillogenic SDS-aS ensembles below 2 mM SDS under conditions very similar to this study. In these complexes aS was described as partially structured and exposing significant hydrophobic surface according to the observed binding of bis-ANS. Giehm et al. [36] also described maximum aS fibrillation around 0.4–0.6 mM SDS. These authors proposed a model in which fibrillogenic complexes are formed by an average of 4 aS molecules associated to a micelle of 40–50 SDS molecules. On the surface of such small micelles, 4 aS molecules are likely too crowded to acquire considerable α-helix structure and therefore remain relatively unstructured. The size of such a micellar complex would be however relatively small compared to the oligomers detected here by DLS. This suggests a self-association of SDS/aS micelles, likely mediated by hydrophobic collapse between unstructured aS regions and possibly involving the non-amyloid component (NAC) region. These clusters would provide a template facilitating intermolecular interactions leading to nucleation of amyloid structure by incorporation of existing aS monomers (Fig 10).

### N-acetylation reduces the rate of amyloid nucleation but PD mutations impair this protective effect against aggregation

In our study N-acetylation did not appear to alter dramatically the conformational- oligomerization transitions in WT aS at low SDS concentrations. We could not detect significant changes in the onset of the formation SDS/aS complexes or in the growth of α-helical structure. However, we observed a clear reduction in the rate of amyloid aggregation induced by SDS as a result of N-acetylation. It is likely that subtle changes produced by N-acetylation on the conformational propensity and dynamics of the aS monomer could make it a poorer substrate for amyloid nucleation, as discussed above. Another possibility is that an increase in alpha-helical propensity at the N-terminal region produced by acetylation may become propagated through the aS chain to the NAC region, enhanced by the interaction with the micelle surface, as observed for small vesicles [55]. A higher affinity for SDS and an enhanced α-helical structure in the SDS-bound state induced by N-acetylation, as reported elsewhere [41], may increase the kinetic barrier of the conformational changes necessary to nucleate amyloid structure within the micellar complexes.

Similarly to the WT variants, the PD variants aggregate mostly under conditions where monomeric aS coexists with a population of the large SDS-aS oligomer complexes. Each PD mutation appears however to favor or disfavor this situation differently, altering the range of SDS concentrations where aggregation occurs. The A53T variants and to a lesser extent the A30P variants aggregate in a broader SDS concentration range than the WT forms. In contrast, the E46K variants aggregate only in a narrow SDS concentration range. The effects of PD mutations in the non-acetylated aS are similar to those observed for aS aggregation catalyzed by surface of small unilamelar vesicles [56]. These effects do not correlate, however, with the overall affinity of each variant for membrane surfaces. The A30P mutation has been reported to decrease aS affinity for anionic phospholipids, whereas the E46K mutation enhances it and the A53T variant shows a similar affinity to the WT form [57]. It is possible that the mutations alter differently the structure and dynamics of the micelle-bound state, as it has been observed for aS interactions with phospholipid vesicles [58], allowing diverse propensities to intermolecular interactions. Nevertheless, N-acetylation does not appear to protect the variants against SDS-induced aggregation as it does for WT aS. This suggests that a long-range cooperative connection between the N-terminal region and the region mediating aggregation may be impaired by the early-onset PD mutations.

Charge removal by N-acetylation in the context of the different PD mutations may also play a role in changing the aggregation propensity but these effects are difficult to envisage in the absence of more precise information about the amyloidogenic aS-SDS micelle clusters. Also, the interactions with the negatively charged SDS head groups may play a determinant role. Different charge-charge interactions might contribute to modify the oligomeric arrangement of aS molecules within these complexes favoring or disfavoring the correct intermolecular contacts for the nucleation process.

### An increase in SDS/aS ratio enhances SDS-aS interactions and reduces aS aggregation

A subsequent rise in SDS concentration above 1.2–1.5 mM produced a gradual decrease in molecular size of the SDS-aS complexes and involves additional growth in the α-helix structure. A higher availability of SDS would probably allow a reduction in the number of aS molecules per micelle [36] allowing and a higher degree of α-helical folding of aS on a larger micelle surface. In the absence of any additional transition, the progressive size decrease would continue up to 10 mM SDS and higher to reach the expected size of a SDS micelle decorated with a single aS molecule, which should be slightly higher than the R_h_ of a SDS micelle (about 2.1 nm). Such behavior is actually observed for the A30P aS variants. In these compact and structured micellar complexes aS becomes protected against aggregation, as observed in all aS variants.

### N-acetylation stabilizes aS-SDS oligomeric complexes with extended α-helical structure

In another transition between approximately 4 and 7 mM SDS, a transient maximum in α-helix structure could also be associated to different oligomerization process, as observed by DLS and DOSY experiments. Contrary to the first one, this transition is considerably enhanced by N-acetylation in the WT protein. Giehm et al. [36] also observed a transient maximum in α-helix, which was attributed to non-fibrillogenic clusters of highly α-helical aS-decorated SDS micelles. Another comprehensive study by CD of the aS-SDS interactions under a variety of pH values and temperatures described a multistate conformational behavior of aS under the effect of SDS binding [50]. In addition to the disordered structure, two partially folded conformations with different α-helical content were proposed, *i*.*e*., a broken-helix horseshoe bent structure, similar to that observed in micelle-bound aS [21], and an elongated helical conformation, such as that observed for aS bound to small unilamellar vesicles [26]. This extended conformation was also described using pulsed dipolar ESR measurements with doubly spin-labeled aS variants [59]. Single-molecule FRET studies using dye-labeled aS confirmed the existence of the micelle-bound bent and extended helical structures occurring at different SDS concentrations [51,60].

Collectively, all this previous evidence in combination with our results indicate that aS can transiently acquire an extended α-helical conformation at SDS concentrations near the CMC of SDS, resulting in micelle crosslinking or micelle remodeling, giving rise to α-helix-rich oligomeric SDS-aS particles (Fig 10). We show here that this transition is enhanced by N-acetylation in WT aS. Assuming that the bent-to-extended conformational transition must involve α-helical folding of the 37–45 segment in the N-terminal domain, there must be some type of long-range connection between this region and the aS N-terminus. This sort of cooperativity appears to be encoded in the aS sequence [39] and would also operate in the initiation and enhancement of membrane binding [37,55].

Many studies have suggested that the actual native functional form of aS is an aggregation-resistant helical multimer [61–63], favored by N-acetylation [40,64] and a yet-to-know additional component, likely of lipid nature [65]. In addition, the equilibrium between aS monomers and aggregation-resistant helical multimers *in vivo* has been associated to a vesicle clustering function of aS [63,66]. These observations may have fundamental consequences to define the role of aS in PD because they suggest that dissociation and unfolding of the helical tetramer could be an obligatory step in the formation of the pathological aggregates [63].

### PD mutations strongly affect the propensity of aS to undergo a bent-to-extended conformational-oligomerization transition

While N-acetylation of WT aS enhances the formation of extended helical oligomers, this effect appears to be disconnected in the PD variants. The two A53T variants could form highly alpha-helical oligomers at high SDS concentrations, independently of N-acetylation. In contrast, none of the A30P variants could undergo this oligomerization transition. The behavior of both E46K aS variants was similar to that of the non-acetylated WT protein but no enhancement of the transition was observed as a result of N-acetylation. These observations are fully consistent with those observed by Ferreon et al. for the non-acetylated PD variants [67]. Our results support the view that the PD mutations may impair the cooperative connection between the N-terminal acetylation and the ability to undergo this conformational-oligomerization transition.

It has been proposed that the switch between the bent and the extended conformation of aS may have a key role for aS function *in vivo* by allowing the protein to bridge between different membranes, thereby regulating synaptic vesicle fusion [59]. aS can induce membrane curvature, budding and tubulation [68] and has been shown to remodel phospholipid vesicles into cylindrical micelles, in which bound aS acquires an extended helical conformation [69]. aS has been also shown to form membrane-bound multimers that promote SNARE complex assembly during vesicle fusion [70] and aS multimerization have been proposed to be implied in the clustering synaptic vesicles, regulating neurotransmitter release [66]. On the other hand, oligomers of aS in a broken helix structure form around small lipid nanoparticles, when incubated at low lipid/protein ratios [71]. This lipoprotein-like function has been related to intracellular transport and metabolism of lipids. All this variety of lipid and membrane interactions are likely to be very finely regulated *in vivo* and involve the concerted action of other proteins. Therefore, besides its importance in initiating and enhancing aS membrane interactions, N-acetylation may have an important role in finely tuning the conformational switch that allows aS to adapt to diverse lipid and membrane contexts related to its function. In this regard, early-onset PD variants may act by deteriorating this connection resulting in diverse deleterious effects under a variety of physiological contexts.

## Supporting information

S1 Fig Determination of the CMC of SDS by light scattering. (TIF)

S2 Fig DOSY determination of the diffusion coefficient of SDS in SDS-aS mixtures. (TIF)

S3 Fig TEM images of SDS-induced oligomers and protofibrils of aS. (TIF)

S4 Fig TEM images of SDS-induced oligomers and protofibrils of Ac-aS. (TIF)

S5 Fig TEM images of SDS-induced amyloid fibrils of aS and Ac-aS. (TIF)

S6 Fig ThT fluorescence kinetics of aggregation of PD variants in presence of SDS. (TIF)
