# Supplementary figures and images for: The influence of N-terminal acetylation on micelle-induced conformational changes and aggregation of α-Synuclein
Source: PLoS One. 2017 May 31;12(5):e0178576. doi: 10.1371/journal.pone.0178576 (PMC5451137; doi:10.1371/journal.pone.0178576)

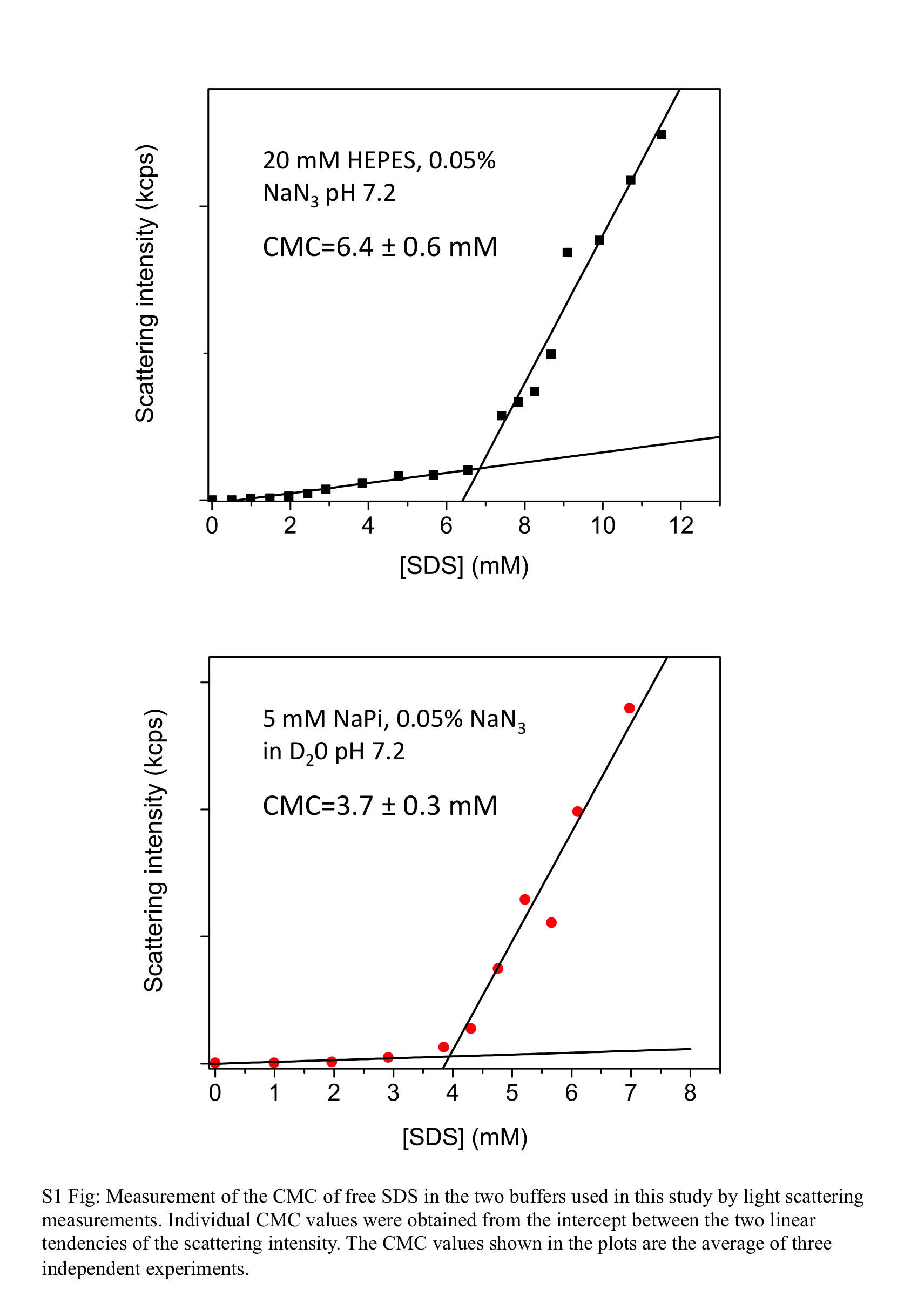

Supplement: S1 Fig — (TIF) [file pone.0178576.s001.tif]

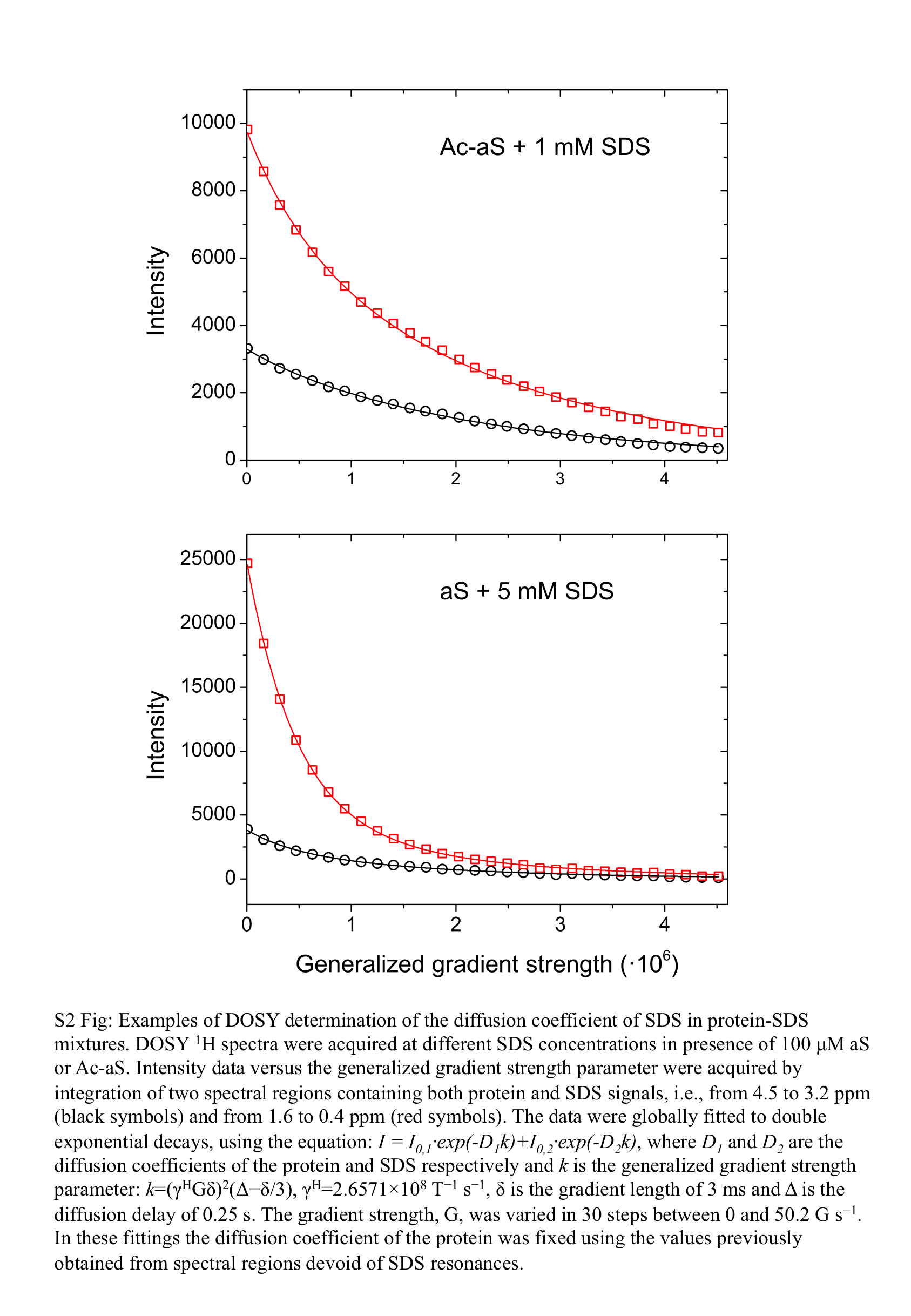

Supplement: S2 Fig — (TIF) [file pone.0178576.s002.tif]

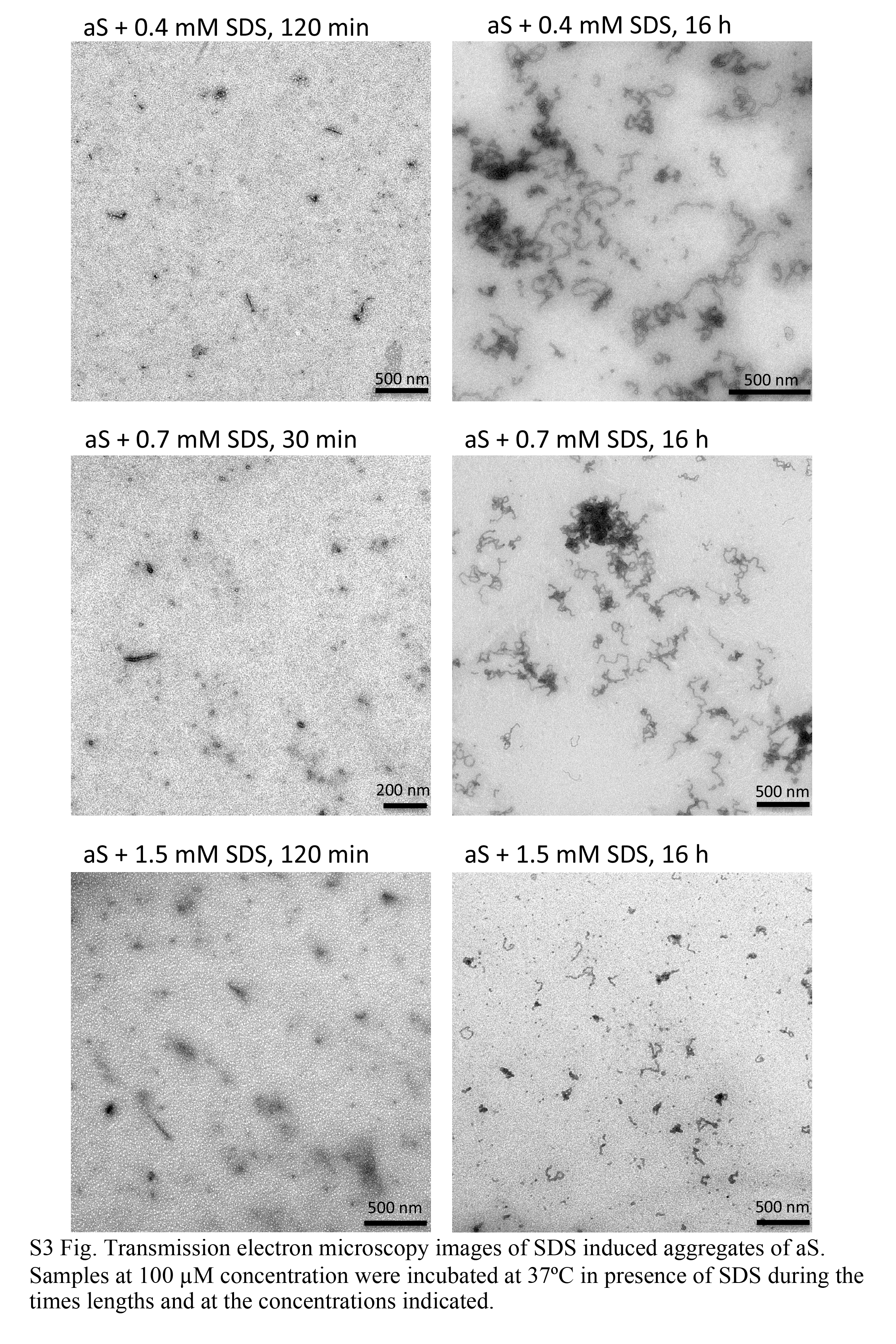

Supplement: S3 Fig — (TIF) [file pone.0178576.s003.tif]

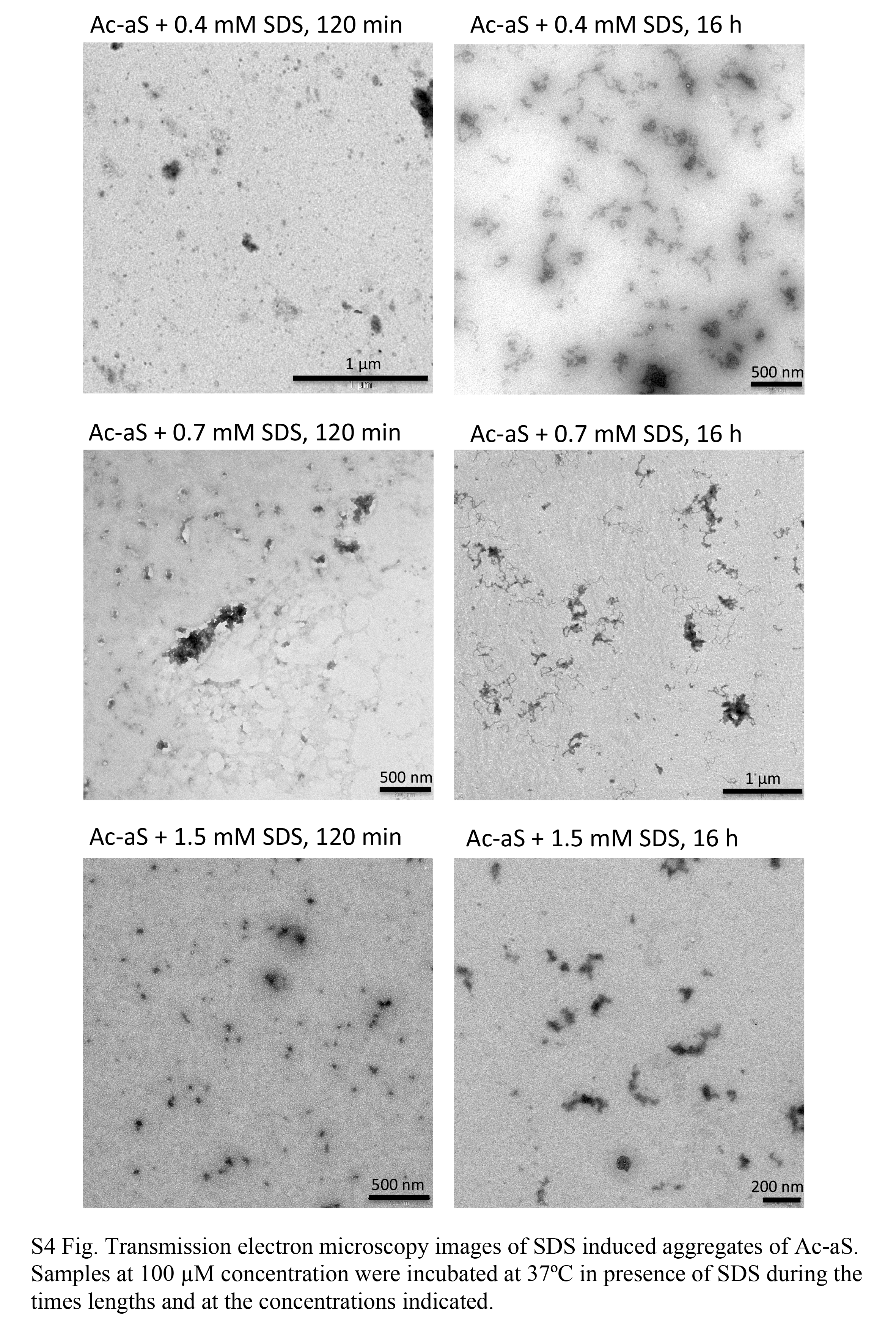

Supplement: S4 Fig — (TIF) [file pone.0178576.s004.tif]

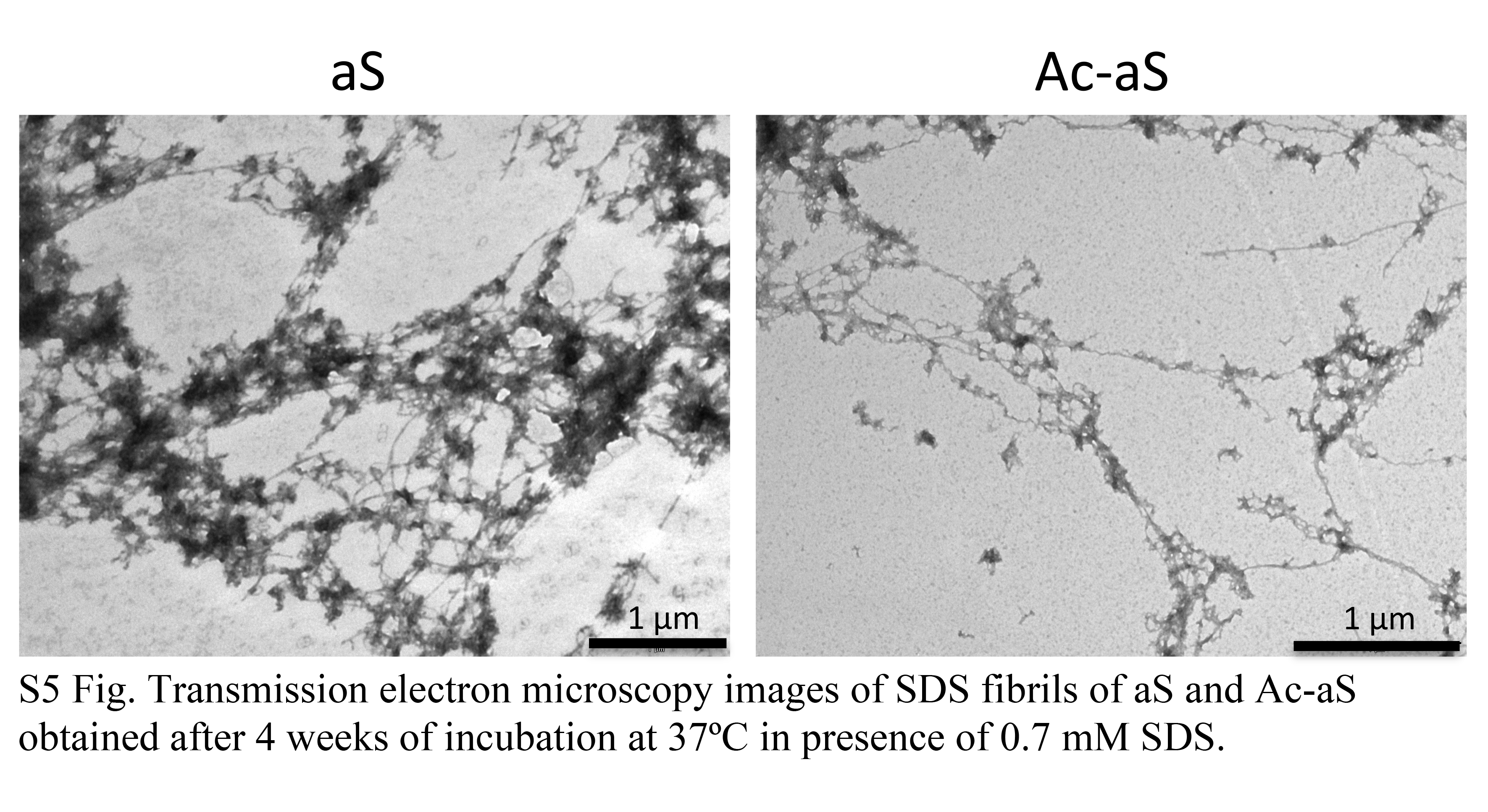

Supplement: S5 Fig — (TIF) [file pone.0178576.s005.tif]

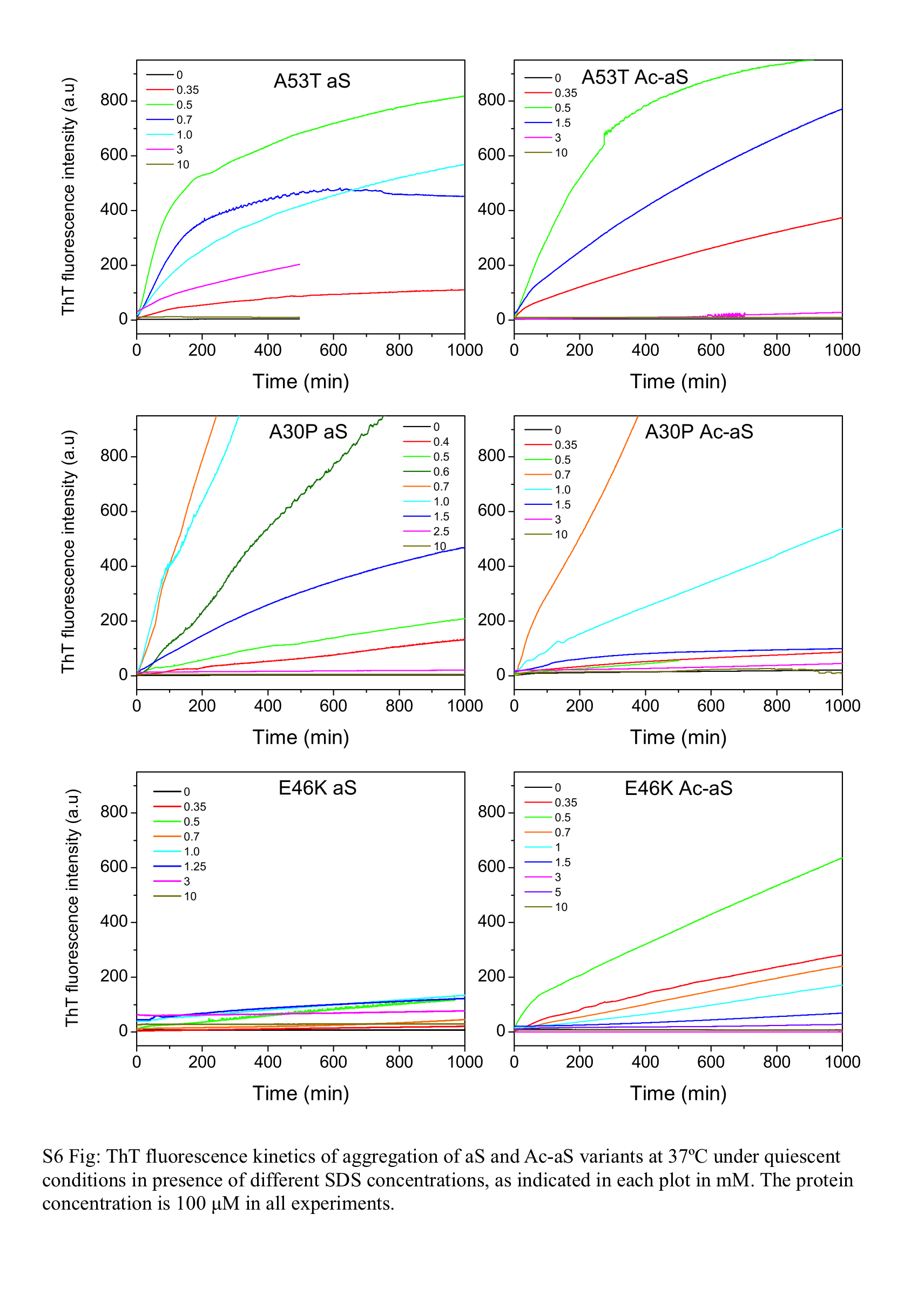

Supplement: S6 Fig — (TIF) [file pone.0178576.s006.tif]
